# Gene regulatory networks on transfer entropy (GRNTE): a novel approach to reconstruct gene regulatory interactions applied to a case study for the plant pathogen *Phytophthora infestans*

**DOI:** 10.1186/s12976-019-0103-7

**Published:** 2019-04-09

**Authors:** Juan Camilo Castro, Ivan Valdés, Laura Natalia Gonzalez-García, Giovanna Danies, Silvia Cañas, Flavia Vischi Winck, Carlos Eduardo Ñústez, Silvia Restrepo, Diego Mauricio Riaño-Pachón

**Affiliations:** 1 Department of Biological Sciences, Universidad de los Andes, Bogotá D.C, Colombia; 2 Department of Design, Universidad de los Andes, Bogotá D.C, Colombia; 3 Regulatory Systems Biology Laboratory, Department of Biochemistry, Institute of Chemistry, Universidade de São Paulo, São Paulo, SP Brazil; 4 School of Agricultural Sciences, Universidad Nacional de Colombia, Bogotá D.C, Colombia; 5 Computational, Evolutionary and Systems Biology Laboratory, Center for Nuclear Energy in Agriculture, Universidade de São Paulo, Piracicaba, SP Brazil

**Keywords:** Information theory, Entropy, Gene regulation, Transcription factors, Biological networks

## Abstract

**Background:**

The increasing amounts of genomics data have helped in the understanding of the molecular dynamics of complex systems such as plant and animal diseases. However, transcriptional regulation, although playing a central role in the decision-making process of cellular systems, is still poorly understood. In this study, we linked expression data with mathematical models to infer gene regulatory networks (GRN). We present a simple yet effective method to estimate transcription factors’ GRNs from transcriptional data.

**Method:**

We defined interactions between pairs of genes (edges in the GRN) as the partial mutual information between these genes that takes into account time and possible lags in time from one gene in relation to another. We call this method Gene Regulatory Networks on Transfer Entropy (GRNTE) and it corresponds to Granger causality for Gaussian variables in an autoregressive model. To evaluate the reconstruction accuracy of our method, we generated several sub-networks from the GRN of the eukaryotic yeast model, *Saccharomyces cerevisae*. Then, we applied this method using experimental data of the plant pathogen *Phytophthora infestans*. We evaluated the transcriptional expression levels of 48 transcription factors of *P. infestans* during its interaction with one moderately resistant and one susceptible cultivar of yellow potato (*Solanum tuberosum* group Phureja), using RT-qPCR*.* With these data, we reconstructed the regulatory network of *P. infestans* during its interaction with these hosts.

**Results:**

We first evaluated the performance of our method, based on the transfer entropy (GRNTE), on eukaryotic datasets from the GRNs of the yeast *S. cerevisae.* Results suggest that GRNTE is comparable with the state-of-the-art methods when the parameters for edge detection are properly tuned. In the case of *P. infestans,* most of the genes considered in this study, showed a significant change in expression from the onset of the interaction (0 h post inoculum - hpi) to the later time-points post inoculation. Hierarchical clustering of the expression data discriminated two distinct periods during the infection: from 12 to 36 hpi and from 48 to 72 hpi for both the moderately resistant and susceptible cultivars. These distinct periods could be associated with two phases of the life cycle of the pathogen when infecting the host plant: the biotrophic and necrotrophic phases.

**Conclusions:**

Here we presented an algorithmic solution to the problem of network reconstruction in time series data. This analytical perspective makes use of the dynamic nature of time series data as it relates to intrinsically dynamic processes such as transcription regulation, were multiple elements of the cell (e.g., transcription factors) act simultaneously and change over time. We applied the algorithm to study the regulatory network of *P. infestans* during its interaction with two hosts which differ in their level of resistance to the pathogen. Although the gene expression analysis did not show differences between the two hosts, the results of the GRN analyses evidenced rewiring of the genes’ interactions according to the resistance level of the host. This suggests that different regulatory processes are activated in response to different environmental cues. Applications of our methodology showed that it could reliably predict where to place edges in the transcriptional networks and sub-networks. The experimental approach used here can help provide insights on the biological role of these interactions on complex processes such as pathogenicity. The code used is available at https://github.com/jccastrog/GRNTE under GNU general public license 3.0.

**Electronic supplementary material:**

The online version of this article (10.1186/s12976-019-0103-7) contains supplementary material, which is available to authorized users.

## Introduction

Generation of new and abundant next generation sequencing data has enabled a better understanding of the molecular dynamics of diseases, and interactions between organisms in general [3, 12, 25, 31, 63]. However, understanding the regulation of transcription in complex systems remains an elusive subject for several reasons. One of the reasons is that experiments to test protein - DNA interactions and their role in regulation are expensive and difficult to replicate [15, 59]. An alternative to experimental approaches to reveal regulator – target interactions is the use of predictive models such as inference of Gene regulatory networks (GRN). GRNs determine the dynamics of transcriptional changes in particular physiological states of an organism, thus playing an important role in understanding the genetic basis of phenotypic traits [28, 41, 42, 64].

Genome-wide clustering of gene expression profiles provides an important first step towards building predictive models by grouping together genes that exhibit similar transcriptional responses to various cellular conditions and are therefore likely to be involved in similar cellular processes [3, 36]. However, the organization of genes into co-expressed clusters provides a very coarse representation of the cellular network. In particular, it cannot differentiate causal interactions from those arising from cascades of transcriptional regulation where many players will have correlated expression without having direct interactions. More generally, as appreciated in statistical physics, long-range order (i.e., high correlation among non-directly interacting variables) can easily result from short-range interactions. Thus correlations, or any other local dependency measure, cannot be used as the only tool for the reconstruction of interaction networks without additional assumptions [27, 65].

In the last decade, several approaches to face these limitations have arisen. The main goal consists on capturing gene interaction as a network model. Nodes of the network are genes, and edges represent direct interactions among genes [4, 17, 35]. In the context of a GRN, these direct interactions represent regulatory events, and thus are causal interactions. The criteria, under which edges are defined, largely vary depending on the methods that are used [44]. Correlation-based models for example, determine these relationships by estimating the linear association of mRNA abundance. This, however, leads to many false positives while discarding non-linear interactions, making these models less likely to provide reliable conclusions on biological problems and undermining the potential uses of expression data altogether. Methods like ARACNE and MRNET use mutual information to capture non-linear dynamics of gene regulation [46, 51, 67], as opposed to methods like BLARS that used penalized linear regression to infer these relationships [26, 56]. Whereas methods such as GENIE3 use machine learning to infer network relationships [30] [29]. More recently developed methods aim at solving the problem on inferring direct gene interaction in gene regulatory networks by exploiting time-series data. For instance, updated versions of ARACNE and GENIE3 have been optimized to deal with this type of data. But also, completely novel approaches like SWING appeared to address the inference of GRNs from time-series data under a Granger causal framework [19]. Transfer Entropy (TE) appears as a way to simultaneously estimate linear and no-linear interactions, which are common in regulatory dynamics, but also as an approach to quantify the time-directed transfer of information between pairs of genes in time-series data [10, 55]. Previous studies have suggested TE as a way to infer GRNs from microarray data but a comprehensive framework is still lacking [53, 60]. Moreover, these approaches focus in few examples of small networks and therefore algorithm performance has little statistical support and is unclear how it might perform in different scenarios with varying network topology [60]. In this study we introduce GRNTE a simple yet comprehensive software implementation to estimate GRN using TE from transcript, or gene expression data.

We benchmarked our newly developed method using the eukaryotic model *Saccharomyces cerevisae*’s GRN. Our benchmarking procedure aims to test our method in multiple sets of data to estimate performance over a range of sub-networks. Subsequently, the method was applied to the plant pathogen *Phytophthora infestans* in a compatible (susceptible host) and incompatible (moderately resistant host) interaction. *Phytophthora infestans,* is the causal agent of potato (*Solanum tuberosum*) late blight disease [21]. This pathogen is a hemibiotroph, meaning that during the beginning of the disease cycle it feeds from living host tissue (biotroph) and later it kills its host and feeds from dead host tissue (necrotroph). A crop plantation may be destroyed in just a few weeks [21]. So far, it is not well understood how and why this transition occurs, from biotroph to necrotroph. Although, effector proteins, that are secreted by the pathogen into the host cell, appear to play a key role [40, 62].

Despite the fact that *P. infestans* is considered a model organism within the oomycetes, and has been depicted as the most destructive pathogen of potato crops [21, 25, 33], the pathogen’s transcriptional dynamics during the interaction with its host are not fully understood [18, 21]. A previous study has provided information on the genes involved in gene expression regulatory functions in Stramenopiles (eukaryotic clade which includes *P. infestans*) [12]. This information can serve as a tool to better understand how the expression of complex phenotypes is regulated in *P. infestans*. Applications of our methodology showed that it can reliably predict where to place edges in the transcriptional regulatory networks and sub-networks. The experimental approach used here provides insights into the biological role of these interactions on complex processes such as pathogenicity.

## Materials and methods

### Model formulation

The model formulation starts considering a GRN with vertices (*v*) and edges (*e*). Then, a probability of interaction for each pair of genes is estimated, by using the marginal probability distribution of each vertex and the joint probability distribution of the pair. In this context, a vertex represents a random variable that corresponds to the expression profile of a gene. Candidate interactions are defined as the mutual information between two gene expression profiles (*I*_*vi*_, *I*_*vj*_). The mutual information for a pair of genes, *v*_*i*_ and *v*_*j*_, is given by *I*(*v*_*i*_, *v*_*j*_) = *H*(*v*_*i*_) + *H*(*v*_*j*_) − *H* (*v*_*i*_, *v*_*j*_), where *H*(*v*_*i*_) and *H*(*v*_*j*_) are the entropy of the *i* th and *j* th gene (vertex), respectively, and *H*(*v*_*i*_, *v*_*j*_) is the joint entropy of *v*_*i*_ and *v*_*j*_ obtained from the joint probability distribution {*p*(*v*_*i*_, *v*_*j*_)} of (*v*_*i*_, *v*_*j*_). Experimental and theoretical approximations to understand gene interactions have used Hill kinetics to model transcriptional interactions [8, 50]. This approach is highly robust when analyzing expression profiles under a myriad of physiological conditions. However, in time variant scenarios, the expression profile is a function of both time (*t*) and the adjacent vertices (see eqs. 1 and 2 in [47].

Given the relationship expressed in eq. 1 in [47] the mutual information of the expression level and time is *I*(*t*, *x*_*i*_)~*H*(*x*_*i*_) as formulated by Frenzel and Pompe [10, 20, 55]. Therefore, to avoid false assignations based on the dependency of two variables with time, we defined the partial mutual information for every pair of genes as done by Frenzel & Pompe [20]:

$$ I\left({v}_{i+l},{v}_j|{v}_i\right)=H\left({v}_{i+l},{v}_i\right)+H\left({v}_j,{v}_{i+l}\right)-H\left({v}_i\right)-H\left({v}_{i+l},{v}_j,{v}_i\right) $$

Where *v*_*i* + *l*_ represents the future values in the *i*th + *l* time of *v*_*i*_. In this expression, *H*(*v*_*i*_) and *H*(*v*_*j*_) have the same values used in the calculation of mutual information but the joint entropy (*H*(*v*_*j*_, *v*_*i* + *l*_)) is different, thus controlling for the unlagged values of the expression profile. This transfer entropy (TE) process corresponds to Granger causality for Gaussian variables in an autoregressive model [7]. However, it also allows the detection of non-linear interactions. We use the framework postulated by Frenzel & Pompe [20] as a stepping stone to estimate interactions between Transcription Factors (TFs).

In accordance with the data processing inequality [10, 37, 60], if two genes *v*_1_ and *v*_3_ interact via a third gene *v*_2_, the mutual information value *I*(*v*_1_, *v*_3_) should be less than min[*I*(*v*_1_, *v*_2_); *I*(*v*_2_, *v*_3_)]. Therefore, for each triplet of genes, direct interactions can be estimated by comparing the values of mutual information and the interaction with minimum value. This is also the case for the TE formulation, where given a lag step *l* the joint entropy *H*(*v*_1_, *v*_3 + *l*_) is under the same constraint. We used this property to avoid estimation of interactions due to spurious events. This differs from Frenzel & Pompe [20] partial mutual information estimation as we exclude effects of third genes without changing our calculation of mutual information. In addition, if an edge is placed between genes *v*_1_ and *v*_2_, the edge has direction *v*_1_ → *v*_2_ if *I*(*v*_1_, *v*_2_) > *I*(*v*_2_, *v*_1_). This process however cannot address bidirectional interactions; thus, the result is a directed network of the genetic interactions based on an expression profile, our implementation also optimizes the lag value (*l*) as it estimates the lag step that maximizes mutual information for each pair of genes.

Transfer entropy takes non-negative values between 0 and infinity. To assess the significance of this measurement we compared the value of each candidate interaction with a null distribution of TE values. For this, we randomly shuffled the expression values of genes across the time series and evaluated the TE for such manifestly independent genes (See next section for the generation of gene expression data). Based on this, we obtained an empirical null distribution of the values of TE. Higher values of TE indicated a stronger relationship. We assigned a *p*-value for each comparison that corresponded to the fraction of TE values that were above or equal to the observed value of TE in the distribution. This was done for 10^5^ different reshuffling iterations in each pairwise comparison to attain reliable estimates of the significance of the interaction. We call this new method Gene Regulatory Networks on Transfer Entropy (GRNTE).

### Yeast network simulated expression data

To evaluate the reconstruction accuracy of our method, we generated several sub-networks from the GRN of the eukaryotic yeast model, *S. cerevisiae* [23]. Using GeneNetWeaver [54], we simulated expression data for 100 sub-networks of *S. cerevisiae*. These networks consist of 200 randomly selected genes. GeneNetWeaver uses ordinary differential equations to simulate expression values, the interaction parameters are estimated based on network topology. We simulated expression values for a time series consisting of 21 points. With these expression data we reconstructed the network topology using GRNTE. For each sub-network, we calculated a receiving operating characteristic (ROC) curve, by estimating the true and false positive rates over a varying threshold and calculated the area under the curve. By doing this we could easily assess the specificity of the algorithm. However, it has been noted that small variations from a value of 1 area under the ROC curve can result in large number of false positives [44]. Therefore, we also assessed the precision and recall (PR) curve and its corresponding area under the curve. Both ROC and PR curves were computed as a measure of the algorithm’s performance. We used R 3.5.1 to carry out all tests of GRNTE. GRNTE requires the libraries “entropy” and “gdata”. We also used the library “igraph” to parse the network objects and to calculate topology metrics. We compared our strategy with five state of the art algorithms: BLARS, dynGENIE3, MRNET, TDARACNE, and SWING. All tests were performed on a single compute node with a single core (2.2 GHz), with 64 GB of available RAM, running on Red Hat Enterprise Linux 6. Each test consumed between 0.5 and 1.0 GB of RAM.

### Evaluation of network properties by assignment of communities

We estimated network modularity by assigning nodes to communities with two different algorithms. Multilevel community detection (MCD) and Markov Clustering (MCL). MCD assigns a community to each mode in the network, so that in the first step there are as many communities as nodes. In subsequent steps nodes are reassigned to a community in a local manner such that it achieves the highest contribution to modularity [9, 38]. Modularity is calculated based on the edge weights (TE values) of incident nodes according to Blondel et al., [9]. Finally, when no nodes can be reassigned to a community (i.e., reassigning a node would rather reduce the overall modularity) each community is considered a vertex on its own, and then the process starts again using that new set of vertices. The final number of communities is determined when the process cannot continue without decreasing the modularity [9]. This algorithm results in assignment of communities in a greedy fashion (i.e., nodes tend to be assigned on communities even if they have few edges). In contrast, MCL assigns communities based on a Markov process [61]. In this algorithm the adjacency matrix (A) is normalized to a stochastic matrix of transition probabilities. The matrix is then squared and normalized iteratively until a convergent state is achieved. In this algorithm a node in row *x* belongs to community with node *y* if the coordinate *A(x,y) = 1* [61]. This results in communities being assigned to a convergent state when nodes share large numbers of edges.

### Selection of differentially expressed genes coding for transcription factors, in *P. infestans*

We decided to apply our model for the reconstruction of part of the regulatory network of the plant pathogen *P. infestans* while interacting with *S. tuberosum*. We determined a set of TFs that were significantly overexpressed during this interaction. Initially, we applied significance microarray analysis (SAM) to determine the set of differentially expressed genes in the available microarray experiment from [16] (GEO accession: GSE33240). We selected the genes with a log2 fold-change (log_2_FC) > 1, and false discovery rate (FDR) q-value ≤0.01. We then cross-validated our results with the Serial Amplification of Gene Expression (SAGE) analysis [3, 24], and chose the TFs that were differentially expressed on both sets of data, according to the criteria mentioned above.

The top 20 differentially expressed genes were selected. These belonged to eight families of TFs (C2H2, DDT, FHA, Jumonji, Myb, Myb-Related, PHD, and TRAF), according to the criteria established in Buitrago-Flórez et al. [12]. All genes associated to these eight families of TFs were selected for further steps. This yielded a total of 54 genes for which we designed RT-qPCR primers. Only 48 of these could be successfully amplified, see below. Subsequently, the 48 genes that could be amplified were selected for the posterior construction and analysis of the transcription regulatory networks.

### Infection assays, RNA extraction, and cDNA preparation

Two cultivars of *S. tuberosum* group Phureja*,* Col2 and Col3, kindly provided by the Potato breeding program from Universidad Nacional de Colombia, were used. Cultivar Col2 is a susceptible variety whereas Col3 is moderately resistant to late blight (C. Ñustez, personal communication). All plants were grown under greenhouse conditions (temperature 18 °C, 12 light hours, and 60% relative humidity).

Leaflets from 6-weeks-old plants were collected and infected with *P. infestans* strain Z3–2 [14]. The strain was grown on Potato Dextrose Agar (PDA) at room temperature (21 °C on average), and a sporangial suspension adjusted to a concentration of 4 × 10^5^ sporangia per ml was prepared as previously described [62]. Infection assays on potato leaflets were performed in moist chambers at room temperature. Ten leaflets were inoculated per time-point and per cultivar, for a total of 60 leaflets per experiment (six time-points per cultivar). Samples were collected every 12 h up to 72 h post inoculation (hpi) and flash frozen in liquid nitrogen. Additionally, we collected the initial inoculum as a reference for the onset of the interaction (0 hpi). This initial inoculum consisted of *P. infestans* growing on PDA medium. The whole experiment was replicated three times (three biological replicates).

Total RNA was extracted using the Qiagen RNeasy extraction kit (Qiagen, Valencia, CA, USA) according to the manufacturer’s protocol and resuspended in 50 μl of RNAse-free water. Treatment with DNAse (Thermo Scientific, Suwanee, GA, USA) was performed to avoid contamination with genomic DNA. Reverse transcription was performed using the DyNAmo 2 step synthesis kit (Thermo Scientific, Suwanee, GA, USA), with 1 μl of RNA in a 50 μl final volume. The oligo-dT were used as primers. Quantification of cDNA was performed using a Nanodrop 1000 (Thermo Scientific, Suwanee, GA, USA), and cDNA was then diluted to a final concentration of 800 ng μl^− 1^ of total cDNA.

### Primer design

We designed primers for reverse transcriptase quantitative PCR (RT-qPCR) using the QuantPrime software [2]. Pairs of primers were designed to span an exon-exon junction to avoid genomic DNA amplification. Primers were tested against a cDNA pool that included all sampling time-points. Primers, which had unspecific amplification or no amplification at all were discarded. A total of 50 primer pairs were kept. Among these, 48 corresponded to TFs and two corresponded to the *Elongation factor 2* and *ß -tubulin* genes, which were used as reference (normalizing) genes for the RT-qPCR. Three different annealing temperatures, 61.5, 60.5, and 59.5 °C, were tested. Among the 48 genes coding for transcription factors, 28 had an optimum annealing temperature of 61.5 °C and 20 had an optimum annealing temperature of 59.5 °C. Therefore, we separated the analyses into two independent groups. Group one corresponded to genes, whose optimum annealing temperature was 61.5 °C and the *ß-tubulin* gene was used as the reference gene (normalizing gene). Group two corresponded to genes, whose optimum annealing temperature was 59.5 °C and the *Elongation factor 2* gene was used as the reference gene. The expected amplicon size was confirmed in an 1.5% agarose gel (Primer sequences available in Additional file 1: Table S1, Evaluation of PCR amplification in Additional file 2: Figure S1).

Gene expression at the different time-points (12, 24, 36, 48, 60, and 72 hpi) was compared to that of sporangia of *P. infestans* growing on PDA medium (0 hpi). Experiments were performed using the Dynamo SyBRGreen RT-qPCR kit (Thermo Scientific, Suwanee, Georgia, USA) according to the manufacturer’s instructions. Samples were run in 96-well plates containing 1 μl of cDNA and a total volume of 10 μl for 40 cycles. Amplification temperature was set according to the annealing temperature for the reference gene in each group of evaluated genes. Expression values were calculated as the relative ratio of expression compared to the reference gene according to Pfaffl method [13, 52].

## Results

### Comparison with existing algorithms

**Fig. 1:**
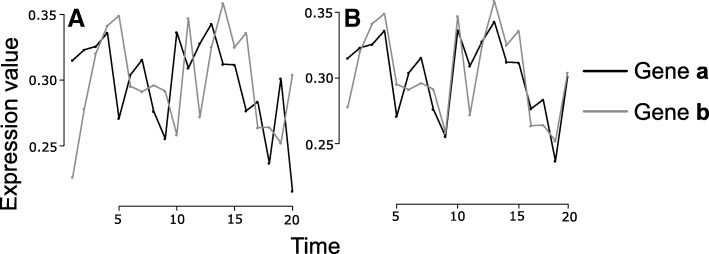
Expression profile from two interacting genes in yeast. Gene **b** regulates gene **a A** Original expression profiles with a mutual information (MI) value of 2.1. **B** When the expression profile of gene **a** is shifted with respect to gene **b**, the MI value increases to 3.4

We evaluated the performance of transfer entropy (TE) on eukaryotic datasets from the GRNs of the yeast *S. cerevisiae.* A total of 100 sub-networks were subsampled consisting of 200 nodes each. For each sub-network we generated time series expression data using GeneNetWeaver [54]. We used this dataset as a gold standard set of verified interactions that would ideally be recovered from the expression data. Based on the expression profiles, we evaluated the TE as the directional increase of mutual information (MI) given by the shifting of the time series (Fig. 1). For each pair of nodes evaluated, a single probability distribution of the values of TE was calculated. The absolute value of TE can be used to describe the weight of the interaction while the *p*-value indicates the significance of the interaction. The shifting of the time series may also give a sense of directionality given that when the MI increases, the regulated TF is shifted with respect to the regulator, and vice versa when the shift occurs the other way around the MI decreases. Using the *p*-values we ranked the regulatory edges from the most confident to the less confident. To evaluate such a ranking independently of the choice of a specific threshold, we used the standard convention of calculating the area under the Precision Recall curve (AUPR) and the area under the receiving operating characteristic (AUROC) [57].

To facilitate comparison between algorithms (i.e. BLARS, dynGENIE3, MRNET, TDARACNE, and SWING, see methods**)**, we transformed the directed graphs generated by the TE to symmetric undirected graphs. Each algorithm assigns a confidence value, between 0 and 1 for each edge. The AUPR determines the proportion of true positives among all positive predictions (prediction precision) versus the fraction of true positives retrieved among all correct predictions (recall) at varying thresholds. Conversely the AUROC estimates the average true positive rate versus the false positive rate.

**Fig. 2:**
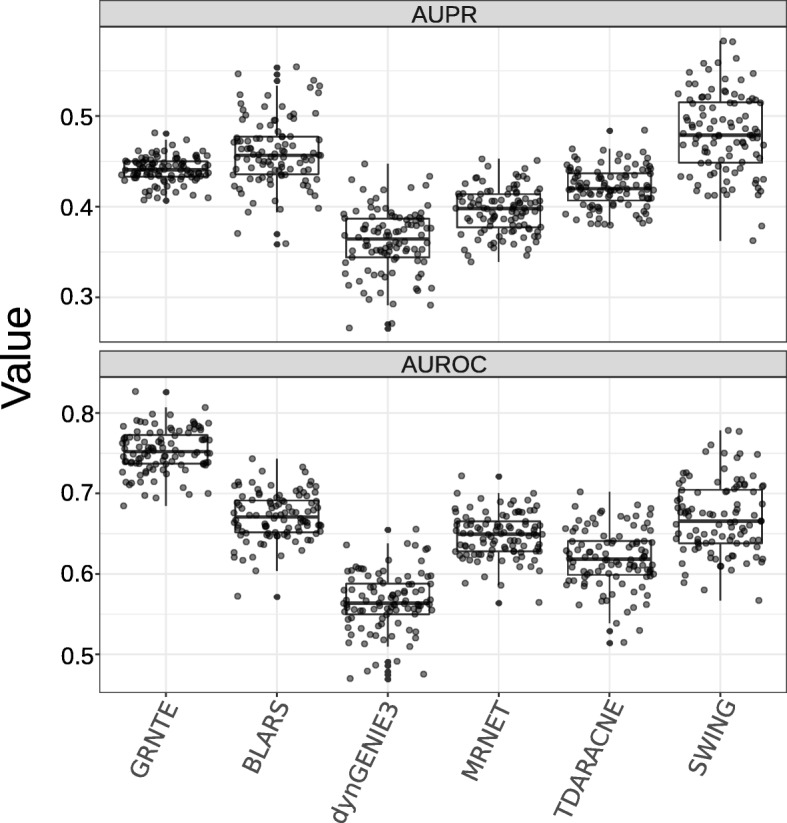
Values of the area under precision recall (AUPR) and the area under the receiving operating characteristic (AUROC) obtained for the benchmark networks of *Saccharomyces cerevisiae*. GRNTE is compared against five methods (BLARS, dynGENIE3, MRNET, SWING, and TDARACNE)

Figure 2 shows the values of the AUPR and the AUROC obtained for the benchmark networks of *S. cerevisiae*, Table 1 shows the average AUPR and AUROC values for a set of 5 networks with 100 genes each used in the DREAM4 challenge. In the benchmark networks GRNTE showed the best performance with respect to the AUROC and the third best performance with respect to the AUPR when compared to the other five methods (BLARS, dynGENIE3, MRNET, SWING, and TDARACNE). For GRNTE, assigned edges have high precision when the confidence threshold is high. However, precision rapidly diminishes when the edges are assigned at lower confidence values, which leads to poor performance in AUPR. BLARS and SWING despite having higher mean AUPR, showed no significant difference from GRNTE and TDARACNE (Pairwise T-test, *p*-value < 0.05). This pattern or high precision at high confidence threshold is preserved when prediction the DREAM4 dataset. Where AUPR is low for all the algorithms. Overall for this dataset, values of AUPR and AUROC are lower than the average obtained in our benchmark networks.

**Table 1:** Average AUROC and AUPR scores of the DREAM4 networks predicted from time series data. The highest score is shown in bold

Algorithm	AUROC	AUPR
GRNTE	0.768	0.410
BLARS	**0.774**	**0.423**
dynGENIE3	0.536	0.228
MRNET	0.638	0.378
SWING	0.657	0.416
TDARACNE	0.591	0.361

AUROC values of GRNTE were significantly higher than most methods tested, which shows a high rate of detection of true positive interactions. This suggests that the GRNTE is more reliable than both TDARACNE and BLARS at high thresholds but rapidly becomes unreliable at low thresholds. Notably although SWING showed a lower mean AUROC it didn’t show any significant differences when compared to GRNTE. These results suggest that the GRNTE may be comparable with state-of-the-art methods when the parameters for edge detection are properly tuned, although it must be noted that the accuracy of GRNTE comes with a higher running time compared to most of the compared methods (Table 2).

**Table 2:** Average CPU time and RAM usage of each algorithm. Each run was carried out in a 200 gene dataset

Algorithm	Average CPU time (seconds)	Average RAM usage (MB)
GRNTE	2813.9	409
BLARS	66.83	321
dynGENIE3	181.03	456
MRNET	715.2	247
SWING	10.11	560
TDARACNE	2977.85	782

**Fig. 3:**
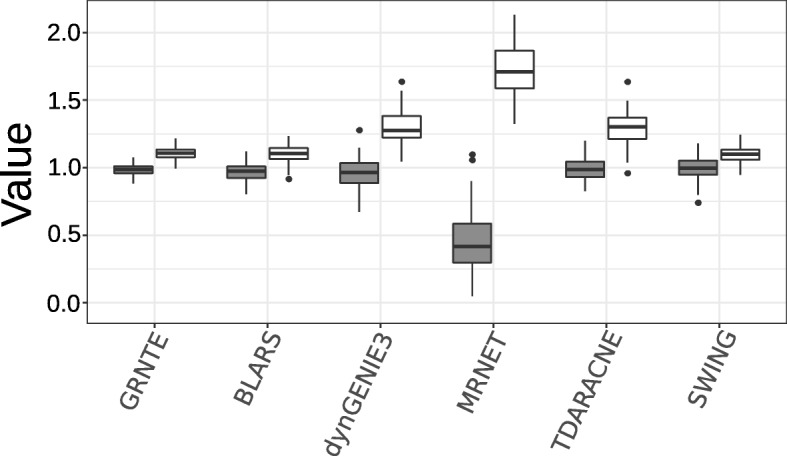
Ratio of the number of communities reconstructed over the number of communities estimated by each algorithm in the gold standard network. Grey is Multilevel community detection (greedy) and white is Markov Clustering (liberal)

Ultimately GRN analysis aims to extract the global structure of a set of gene interactions [6, 38, 48], using modularity as a measurement of structure, we used the benchmark dataset as a mean to recover the network structure. We calculated the number of communities in each of the sub-networks of the dataset. We used a conservative algorithm (MCL) and a greedy algorithm (MCD) for calculation of the number of communities. We calculated the ratio of the number of communities reconstructed over the number of communities estimated by each algorithm in the gold standard network (Fig. 3). GRNTE preserves community structure as the mean ratio is close to one both in the conservative and the greedy algorithm (1.07 and 1.10), whereas the other algorithms deviated from this metric. Indicating that although a given algorithm may show a low number of spurious edges, in most cases, the spurious edges contribute to misleading clustering which can be detected by a multilevel community detection as in the case of MRNET. Additionally, algorithms like TDARACNE and dynGENIE3 lack important edges which result in the constitution of several small clusters that misrepresent the network structure, as seen by MCL clustering. BLARS and SWING showed similar ratios to those of GRNTE, which reveal its reliability at estimating network structure.

### Application of transfer entropy to the *P. infestans* dataset

**Fig. 4:**
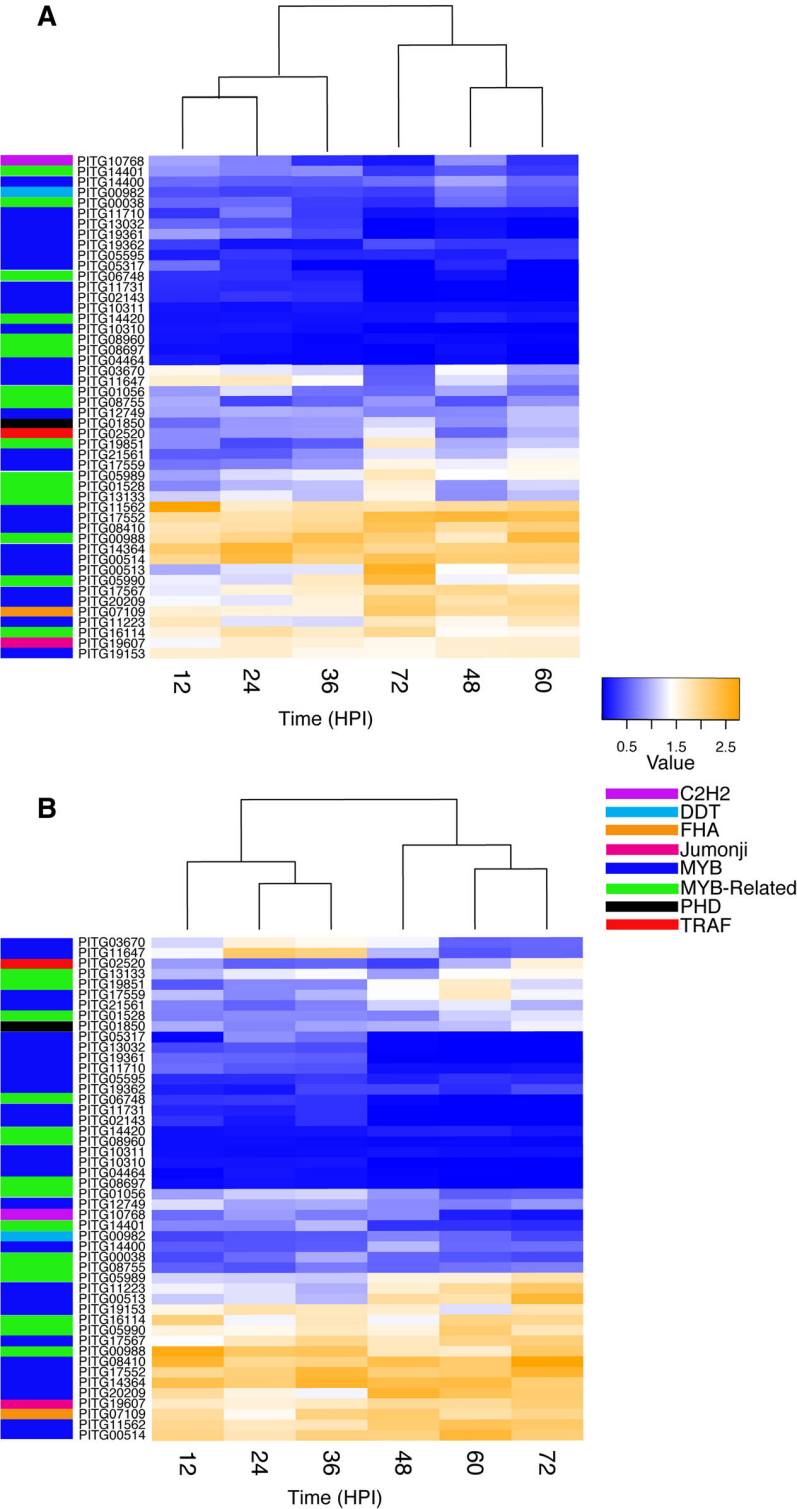
Expression profiles for 48 transcription factors (TFs) in *Phytophthora infestans* obtained by RT-qPCR during the infection process in *Solanum tuberosum* group Phureja cultivars **a** Col2 (susceptible) and **b** Col3 (moderately resistant)*.* Expression values are shown for down-regulated genes in blue and up-regulated genes in orange. Expression ratios are calculated relative to time 0 hpi (*P. infestans* growing on potato dextrose agar (PDA) medium). Hierarchical clustering shows two distinct groups during the infection of the leaf tissue corresponding to the biotrophic (12, 24, and 36 hpi) and necrotrophic (48, 60 and 72 hpi) phases, respectively. The names of the TF families are also denoted

The expression profiles of 48 TF genes of *P. infestans* during its interaction with potato cultivars Col2 and Col3 were assessed via RT-qPCR. An expression profile was constructed for each TF by calculating the ratio of the expression for the gene at each time-point after inoculation in comparison with the expression of the same gene in *P. infestans* growing in PDA medium (Time 0) (Fig. 4; Additional file 3: Table S2). Hierarchical clustering showed that the expression of the genes at 12, 24, and 36 hpi (when the pathogen is growing biotrophically) grouped separately from that at 48, 60, and 72 hpi (when the pathogen grows as a necrotroph, killing the host tissue), for both the moderately resistant and susceptible potato cultivars (*p*-values of clustering were 0.03 and 0.06 for Col2 and Col3 respectively) (Fig. 4). When infecting the Col2 cultivar (susceptible), a total of 21 and 15 TF genes were consistently down-regulated and up-regulated, respectively. In the case of Col3 (moderately resistant) 23 and 16 *P. infestans* genes were consistently down-regulated and up-regulated, respectively.

When comparing the expression profiles of the *P. infestans* genes between the two cultivars we did not observe major changes (Additional file 4: Figure S2). In contrast, in both cultivars about 23% of the genes studied showed a drastic change in expression during the time series (measured by series autocorrelation). In both cultivars, genes PITG_03670 and PITG_11647 (both annotated as hypothetical protein with a Myb domain) had a significant transition from high expression to low expression. Whereas genes PITG_01528 (cell division cycle 5-related protein) and the Myb-like DNA-binding proteins, PITG_05989, PITG_11223, PITG_13133, PITG_17559, PITG_19851, and PITG_21561, displayed a transition from low expression to high expression (according to Durbin Watson test, *p*-value > 0.05). Notably genes PITG_01528, PITG_11223, PITG_13133, PITG_19851, and PITG_21561 only exhibited this pattern in cultivar Col3. Additionally, gene PITG_00513 (cell division cycle 5-related protein) had a different expression pattern in Col2, where it went from highly expressed at the early stages to lowly expressed at the late stages (Fig. 4).

**Fig. 5:**
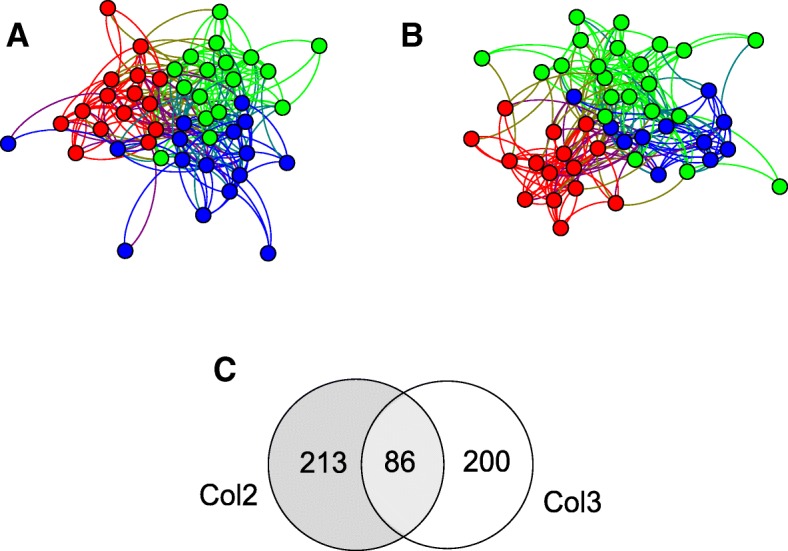
Regulatory networks for *Phytophthora infestans* infecting *Solanum tuberosum* group Phureja leaflets. Three communities from susceptible and resistant cultivars Col2 and Col3 are depicted. **a** The Col2, susceptible cultivar, network had 299 edges and a modularity value of 0.2878. **b** The Col3, resistant cultivar, network had 286 edges and a modularity value of 0.3177. All nodes from community 1 (red), 17 nodes from community 2 (green), and 11 nodes from community 3 (blue) were shared between the two networks. Five nodes from community 3 in Col2 were assigned to community 2 in the Col3 cultivar. **c** Common edges between Col2 and Col3 regulatory networks

The transcript expression time series was used to infer the regulatory network in both cultivars (Fig. 5). A total of 299 edges were identified for the *P. infestans* regulatory network when infecting Col2 (Col2 network; Additional file 5: Network S1) and 286 edges when infecting Col3 (Col3 network; Additional file 6: Network S2). The Col2 network had an average degree of 12.45, not different from an Erdos-Renyi random network with the same number of nodes and an average number of edges (*p*-value = 0.32932). This network was composed of 3 modules as detected by MCD with a modularity value of 0.2878 (Fig. 5A). The Col3 network showed an average degree value of 11.96 (p-value = 0.38011). There was no observable correlation between expression level and node degree (Additional file 7: Figure S3). We found three communities as well in this network with a modularity value of 0.3177 (Fig. 5B). A total of 86 common edges were found between these two networks (Fig. 4C; Additional file 8: Network S3). A high level of rewiring was observed in both the Col2 and the Col3 networks, with a Hamming distance of 318. That is, 318 edge addition or removal operations were required to convert one network into the other. However, this number is significantly less than the hamming distance between two random networks according to the Erdos-Reyni model with the same number of edges (p-value = 0.00094). Both networks had the same number of nodes; therefore, the same level of rewiring inside the sub-network.

**Fig. 6:**
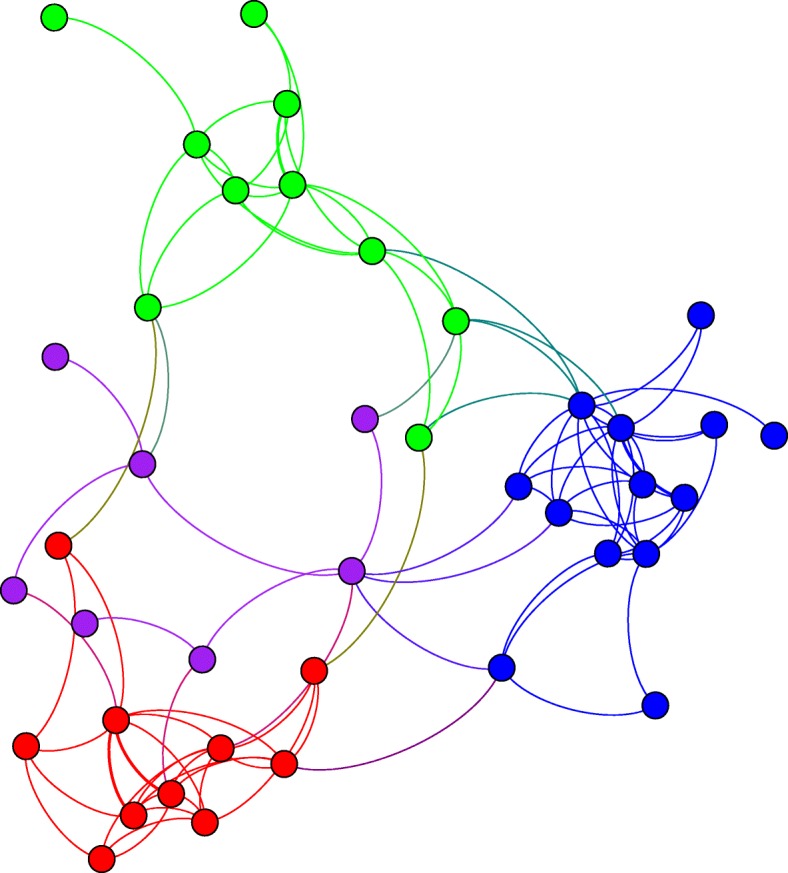
Intersection network for the cultivars Col2 and Col3. Four communities were found, the communities in red, green, and blue were composed by the same nodes in the Col2 and Col3 networks. The fourth community (purple) is composed by genes that showed consistent up-regulation in the Col2 and Col3 hosts

To further evaluate the similarities between cultivars Col2 and Col3, we assessed the intersection between the two inferred networks (Fig. 6). For this, we selected the common edges between the two sub-networks and the nodes associated to these. This was considered the shared sub-network and consisted of a small network of 40 nodes and 86 edges with an average degree of 4.25. We found a total of 4 communities in this network, three of this communities where composed of nodes similar to those found in the communities identified in Col2 and Col3 network reconstructions, A fourth community however was composed of nodes belonging to all three communities (Fig. 6).

## Discussion

Network analysis is a valuable approach to understand biologically relevant phenomena as well as formulating hypotheses to be tested in the laboratory [5, 6, 58]. These networks serve as a basis for the creation of models of physiology at a cellular scale. In order to obtain robust models, it is necessary to address the challenges of reconstruction from empirical data to make accurate predictions and advance our understanding of biologically relevant phenomena [27, 28]. Here we presented an algorithmic solution to the problem of network reconstruction in time series data. This analytical perspective makes use of the dynamic nature of time series data as it relates to intrinsically dynamic processes such as transcription regulation, were multiple elements of the cell (e.g. transcription factors) act simultaneously and change over time. Thus, understanding the relationships of these changing elements can give insights of the basic biology of complex phenomena such as disease.

Like similar algorithms used for the purpose of GRN reconstruction, our strategy attempted to discern connections between genes via establishing correlations within their expression profile. It however differentiates from methods such as LASSO or LARS as it evaluates non-linear relationships using MI (Mutual Information) [29, 56]. Some other methods implement MI to establish edges between nodes; these however only evaluate the relationship in a static manner [46]. We introduced a dynamic aspect by considering that regulators and regulated genes have a shift in their expression profile. Thus, we reduced sources of noise in the assignation of confusing edges between nodes, by having the same parent (i.e. the node controlling both nodes) while at the same time assigning directionality to the edge. We also provide a way to score the edges that do not depend on the empirical ranking of the MI values. Our in-silico results suggest that including time into the prediction of edges results in a high number of predicted edges. Although algorithms like SWING incorporate time delays into their architecture, and it shows high performance, the use of linear models may inhibit detection of some edges that follow non-linear dynamics. The incorporation of multiple strategies into an algorithmic pipeline has been noted in the past. Marbach et al., [43] note that incorporating algorithms with different operating principles (e.g., MI, and regression) results in higher performance overall as false positive edges are weighted out by agreement between algorithms, and rare edges can be detected by incorporating multiple avenues. Therefore, incorporating methods such as BLARS, SWING and GRNTE may have advantages in network analysis of transcriptomic data as this rely in different principles, and can altogether overcome the weaknesses of each individual approach.

Another significant concern is the validation of the resulting network. A standard framework has been set up by DREAM in order to compare different algorithms [45, 54], the incompleteness of gold-standard networks remains a demanding challenge nonetheless. Missing edges in a gold-standard network can lead to the underestimation of true positives as these mask as false positive results. As further research adds more edges to the gold standard network, the predicted true positives can either increase (i.e., false positives could decrease) or remain constant. This is because the number of predicted positive edges is the sum of the number of true positives and the number of false positives [4, 44]. The lack of well-curated gold-standard networks causes biases in the measurement of algorithmic performance. For this reason, simulated data is often preferred to test network reconstruction in addition to validating the reconstruction on biological networks. The assessment of the performance of different algorithms on real biological networks will improve soon as evidence for more gold-standard edges is gathered. In the interim, synthetic networks will complement the algorithm benchmarking experiments. Therefore, it is crucial to use GRN simulation tools that account for as many biological factors as possible [54]. In addition to benchmarking procedures should include large number of different networks, as network topology has large effects on algorithm performance as evidenced by the large variances in AUPR and AUROC values displayed in all algorithms. Moreover, in cases where a small set of networks is present this can lead to overestimate or underestimate the predictive quality of a given algorithm. For example, the above-average scores we obtained in our benchmarking setup compared to those available for DREAM4. These datasets proved to be challenging to all the algorithms and overall could lead to the conclusion that the algorithms have low performance when in another set of networks, the algorithms may have shown higher competence.

The use of GRN simulation tools becomes particularly relevant when one intends to evaluate the network structure as a whole. If the objective is to understand physiology as an emergent property of gene expression, properly assessing the network features is paramount to make reliable predictions and design constructive experiments [6, 42, 49]. We have shown that although not all the edges inferred in a network are accurate, it is still possible to confidently estimate global properties of the network such as modularity. We show that these properties tend to be preserved even if the inference of edges is not completely accurate as variation in recall is not reflected in variation of community detection. If the properties of the network can be faithfully reconstructed without fully assessing the individual edges, a robust transition from simulated datasets into experimental ones can be made based on the assumption that the noise of missing and spurious edges is balanced. It is therefore necessary to consider the type of experiments in which each algorithmic solution can be used, whereas approaches like BLARS and ARACNE are useful in transcriptome assays of static physiological states [39, 40], alternatives like GRNTE, SWING or TDARACNE are shown to be a better alternative for time series data.

We have shown that network inference from expression data is a key tool for improving the biological insights obtained from transcriptomics data. Exploiting time series transcriptome analyses has helped in the understanding of the infection process of animal pathogens. Such studies have shown, for instance, that in *Plasmodium falciparum* distinct clusters of genes have a differential behavior during the different stages of the complex life cycle of this human pathogen [11]. However, in *P. infestans*, expression profiling did not reflect synchronized changes in time as it was observed in *P. falciparum* phaseograms, thus rendering difficult the study of physiological changes of the infection stages of *P. infestans’* life cycle. Notably, most of the genes sampled in this study, showed a rather drastic transition from growing on artificial-medium (0 hpi) to growing on leaf tissue. However, during leaf infection, from 12 to 72 hpi drastic transcriptional changes did not occur. Despite having a few variations throughout the expression profile, hierarchical clustering of the expression data discriminated two distinct periods during the infection: from 12 to 36 hpi and from 48 to 72 hpi. These distinct periods can be associated with two phases of the life cycle of the pathogen when infecting the host plant: the biotrophic and necrotrophic phases. Transcription factors within the GRNs changed their expression levels and gained or lost interactions throughout the infection process. This reflects the role of TFs in controlling different aspects of the infection process despite showing only slight changes in their expression level. When comparing the transcriptional patterns between the two cultivars, again, very few genes were differentially expressed. Most of these genes were annotated as Myb -like DNA-binding proteins. The role of the Myb transcription factor during early infection of *Phytophthora sojae* was demonstrated by Zhang et al. [66], where the loss of *PsMYB1* resulted in abnormal sporangial development and affected zoospore-mediated plant infection. More studies on the role of Myb transcription factors on the biology of infection of *P. infestans* are needed to understand the tight transcriptional control of a compatible and incompatible interactions.

On the other hand, the networks allowed us to evaluate aspects of transcription, which are beyond the raw expression changes as was shown when exploring the changes in gene expression using the GRN in each environment/host. As mentioned above, the most significant changes in the expression values for most of the TFs were observed between the oomycete growing in culture medium and *in*-*planta* but differences in the expression ratios of the TFs of the pathogen when infecting Col2 or Col3 were not significant. However, when using the GRNs, for example, highly connected nodes, and gene modules in the GRNs did not necessarily agree with drastic changes in expression profiles, thus highly expressed genes do not necessarily have high centrality and hierarchical clustering groups of genes do not correspond to network communities. Additionally, genes that show changes in expression in different hosts do not show highly different centrality. Our comparison of the two networks, showed that despite having small changes in gene expression, a high number of changes occurred in the establishment of connections inside the GRN for each host. The fact that only about 30% of the interactions of one network were preserved in the other network, suggest that the system shows several changes comparing a compatible and an incompatible interaction. Although the number of modifications was much less than expected between two random networks, it is possible to speculate that the rewiring of *P. infestans* GRN is subjected to several constraints and that the process has been evolutionarily optimized. If we consider that any operation of rewiring is possible, the expected value for the Hamming distance would be very close to those of two random networks. However, the control of the transcription regulation is not random, as this value is much lower. Editions to the network structure, although many, should be precise to keep the balance and functionality of the network [4]. It is important to note that these differences are not seen when observing the raw expression values directly and that through network reconstruction it is possible to establish differences in the infection process in the two different hosts.

At the same time, preserved topological features (such as modularity and the large fraction of genes which remain affiliated to a community) indicate that there are core regulatory functions preserved between two different environments. Thus, there is a tight control in the regulation of the transcriptional program in a compatible and incompatible interaction. Just a relatively small subset of changes is required to have a completely different behavior, compatible (Col2) vs incompatible interaction (Col3), without drastic changes in TF expression levels, compared to the random case. Large differences in expression levels in one gene may be balanced by smaller changes in other components in the GRN. However, our reconstruction was not able to distinguish rearrangements occurring at higher levels in the whole GRN. A larger sample of genes is needed to search for evidence that may support larger transcriptional rewiring.

Community organization has been proposed as a property indicative of functional units in complex networks [22, 58]. Our analysis of the modular organization of the networks showed that different modules are highly conserved. This suggests that a small rewiring of the regulatory network could have a large impact on the functional organization of the network [22, 38]. Our results on the intersection of the two cultivars’ networks showed the presence of a fourth community. This could indicate the presence of core circuits on the GRN since these circuits are very active transcriptionally during the infection process. Testing the functional activity of these genes should be of primary importance, as these may play an important role in the stability of the network and information flow between different higher-level modules. These modules could be responsible for interaction compatibility, as the pathogen tends to preserve these even after heavy rewiring. The effect of plant resistance may be better understood as a network rewiring. The effect of incompatibility (plant resistance) may be better understood as a network rewiring. The ‘rewired’ genes, may be targeted in the early stages of infection by the pathogen. If this control was exerted at the protein interaction level, it would not be detected at the mRNA level. As a response, the pathogen may shift the regulatory interactions of these genes while keeping a functional structure. The genes that show variation among the different modules may act as the emissaries of the transcriptional state of the plant and thus, could prove to be of high interest.

Expression profiling of *P. infestans* has been helpful in the discovery and characterization of the effector genes and in distinguishing between different stages of the infection [32, 16]. Also, transcriptomic studies have helped to determine particular genes involved in host defense suppression as well as control of internal signaling [34]. However, there is still a major barrier to efficiently assess the pathogenic behavior of *Phytophthora*, and to fully understand phenomena such as host specificity or hemibiotrophy. Network biology proposes that data coming from large experiments can be analyzed in several different layers. A regulatory network built from transcriptional data may be interpreted from its basic properties to more complex levels all of which may give different insights depending on the context [5, 6, 22, 58]. We have shown that subtle changes in transcript abundance, do not necessarily point to high levels of similarity on the network level. The topological properties of the network may prove to be a better point of comparison for datasets in which conventional analysis may not yield high differences.

Complex behavior such as hemibiotrophy, may be explained via the effect of regulatory events occurring at distinct times. The regulatory capacities of the TFs inside a network may be best explained by the information that these transmit to other elements of the network. Small differences in network rewiring and conserved levels of expression, may be explained by the effect of each individual TFs, in terms of its information flow inside the network. The information flow can be assessed by estimating the betweenness centrality; genes PITG_10768 (zinc finger C2H2 superfamily) and PITG_08960 (Myb-like DNA binding protein) showed the highest betweenness centrality in Col2 and Col 3 sub-networks respectively. These genes are constantly down-regulated and this agrees with the hypothesis that shifts in physiological behavior are controlled via negative regulation in *Phytophthora* [34, 40]. These nodes, with high betweenness centrality, have a high influence over the network, as shown be simulation of an infection process [39]. If the activation of a physiological state is mediated by the selective shut down of particular transcription factors, then particular regulators may be acting in each case to control the response to different environments.

The preservation of modules, despite heavy rewiring of the network, may indicate that these circuits have large biological importance and play key roles in the physiology of infection. In organisms such as *P. infestans*, analytical tools that elucidate the process via study of the mRNA, can be greatly expanded via network reconstruction. Using this framework, differences in the behavior of an organism in different environments can be found, as shown in the rewiring for the sub-networks in different environments. Additionally, although expression profiling may be a powerful tool to determine major genes involved in the infection process, it is limited to clearly discriminate possible mechanism and hypothesis underlying host-pathogen interactions, network analysis broaden the analytical power of this data sets as it allows to determine modules and to narrow the number of candidate genes for experimental validation [5]. Unlike organisms like *P. falciparum* [11], gene expression changes in *P. infestans* are less directly indicative of regulatory function changes. This is the first study to use network reconstruction as a way to overcome the limitations of gene expression profiling. Some of the ideas discussed here are widely used in other fields [1, 6, 22, 39] and the incorporation of these tools into the study of plant-pathogen interactions can open a window to better understand the behavior of pathogens and to propose effective alternatives for their control.

## Conclusions

Here we presented an algorithmic solution to the problem of network reconstruction in time series data. This analytical perspective makes use of the dynamic nature of time series data as it relates to intrinsically dynamic processes such as transcription regulation, where multiple elements of the cell (e.g. transcription factors) act simultaneously and change over time. We applied the algorithm, GRNTE, to study the regulatory network of *P. infestans* during its interaction with two hosts which differ in their level of resistance to the pathogen. Although the gene expression analysis did not show differences between the two hosts, the results of the GRN analyses indicated rewiring of the genes’ interactions according to the resistance level of the host. This suggests that different regulatory processes are activated in response to different environmental cues. Applications of our methodology showed that it could reliably predict where to place edges in the transcriptional networks and sub-networks. The experimental approach used here can help provide insights on the biological role of these interactions on complex processes such as pathogenicity. The code used is available at https://github.com/jccastrog/GRNTE under GNU general public license 3.0.

## Additional files

Additional file 1: **Table S1.** Primer sequences for TFs genes in *P. infestans* assayed in this study. Forward and reverse primers were designed by QuantPrime [2]. Expected annealing temperature is also shown. (XLSX 12 kb)

Additional file 2: **Figure S1.** PCR amplification for testing primer viability. cDNA extracted from *P. infestans* in PDA media was amplified at 45 PCR cycles. Observed fragment size x to expected fragment size (~ 50-70 bp) when observed in 2% agarose gel. Some primer dimers can be observed. (PDF 387 kb)

Additional file 3: **Table S2.** Mean expression values for 48 TFs from *P. infestans* during the interaction with *S. tuberosum* group Phureja Col3 and Col2. Relative expression is calculated comparing RT-PCR measurements to time 0 h.p.i. for both Col2 and Col3. Expression profiles are also compared between the two hosts to observe differences during the interaction specific of cultivar. (XLSX 26 kb)

Additional file 4: **Figure S2.** Expression profile of Col2 compared to Col3**.** Heatmap representing the expression profiles were compared for the two cultivars. For each transcript each time point is compared to the same timepoint in the other cultivar (e.g., Col2 PITG_05317 12 h.p.i. is compared to Col3 PITG_05317 12 h.p.i.) Although only minor changes in expression are observed Genes overexpressed in Col2 and a clear separation between early and late infection can be observed by hierarchical clustering. (PDF 189 kb)

Additional file 5: **Network S1.** Network file for the sub-network of *P.infestans* when interacting with *S. tuberosum* Col2, as observed in Fig. 5. Network in XML format, can be visualized in Cytoscape. (XML 28 kb)

Additional file 6: **Network S2.** Network file for the sub-network of *P. infestans* when interacting with *S. tuberosum* Col3, as observed in Fig. 5. Network in XML format, can be visualized in Cytoscape. (XML 27 kb)

Additional file 7: **Figure S3.** Expression is unrelated to degree. Node degree is computed for each node in the network and plotted against it mean expression value in Col2 (A) and in Col3 (B). No correlation is observed. (PDF 179 kb)

Additional file 8: **Network S3.** Network file for the subnetwork of *P. infestans* extracted from the intersection of nodes and edges between networks for infection in Col2 and Col3 as observed in Fig. 6. Network in XML format, can be visualized in Cytoscape. (XML 16 kb)

## Abbreviations

GRN: Gene regulatory networks
GRNTE: Gene Regulatory Networks on Transfer Entropy
Hpi: Hours post inoculum
TE: Transfer entropy

## References

[1] Albert R, Barabási A-L (2002). Statistical mechanics of complex networks. Rev Mod Phys.

[2] Arvidsson S, Kwasniewski M, Riaño-Pachón DM, Mueller-Roeber B (2008). QuantPrime--a flexible tool for reliable high-throughput primer design for quantitative PCR. BMC Bioinformatics.

[3] Avrova AO, Venter E, Birch PR, Whisson SC (2003). Profiling and quantifying differential gene transcription in Phytophthora infestans prior to and during the early stages of potato infection. Fungal Genet Biol.

[4] Babu MM, Luscombe NM, Aravind L, Gerstein M, Teichmann S a (2004). Structure and evolution of transcriptional regulatory networks. Curr Opin Struct Biol.

[5] Barabási A-L (2011). The network takeover. Nat Phys.

[6] Barabási A-L, Oltvai ZN (2004). Network biology: understanding the cell’s functional organization. Nat Rev Genet.

[7] Barnett L, Barrett AB, Seth AK (2009). Granger causality and transfer entropy are equivalent for gaussian variables. Phys Rev Lett.

[8] Bhaskaran, S., Umesh P, & Nair, A. S. (2015). Hill equation in modeling transcriptional regulation. In Systems and synthetic biology (pp. 77–92). Dordrecht: Springer Netherlands.

[9] Blondel VD, Guillaume J-L, Lambiotte R, Lefebvre E. Fast unfolding of communities in large networks. J Stat Mechanics. 2008:6.

[10] Bossomaier T, Barnett L, Harré M, Lizier JT (2016). Transfer Entropy. An introduction to transfer entropy.

[11] Bozdech Z, Mok S, Hu G, Imwong M, Jaidee A, Russell B (2008). The transcriptome of plasmodium vivax reveals divergence and diversity of transcriptional regulation in malaria parasites. Proc Natl Acad Sci U S A.

[12] Buitrago-Flórez FJ, Restrepo S, Riaño-Pachón DM (2014). Identification of transcription factor genes and their correlation with the high diversity of Stramenopiles. PLoS One.

[13] Bustin SA, Benes V, Nolan T, Pfaffl MW (2005). Quantitative real-time RT-PCR--a perspective. J Mol Endocrinol.

[14] Céspedes MC, Cárdenas ME, Vargas AM, Rojas A, Morales JG, Jiménez P (2013). Physiological and molecular characterization of Phytophthora infestans isolates from the central Colombian Andean region. Revista Iberoamericana de Micologia.

[15] Chu D, Zabet NR, Mitavskiy B. Models of transcription factor binding: sensitivity of activation functions to model assumptions. J Theor Biol. 2009. 10.1016/j.jtbi.2008.11.026 19121637

[16] Cooke DEL, Cano LM, Raffaele S, Bain RA, Cooke LR, Etherington GJ (2012). Genome analyses of an aggressive and invasive lineage of the Irish potato famine pathogen. PLoS Pathog.

[17] Davidson E, Levin M (2005). Gene regulatory networks. Proc Natl Acad Sci U S A.

[18] Ewig EE, Šimko I, Smart CD, Bonierbale MW, Mizubuti ESG, May GD, Fry WE (2000). Genetic mapping from field tests of qualitative and quantitative resistance to Phytophthora infestans in a population derived from Solanum tuberosum and Solanum berthaultii. Mol Breed.

[19] Finkle JD, Wu JJ, Bagheri N (2018). Windowed granger causal inference strategy improves discovery of gene regulatory networks. Proc Natl Acad Sci.

[20] Frenzel S, Pompe B (2007). Partial mutual information for coupling analysis of multivariate time series. Phys Rev Lett.

[21] Fry W (2008). Phytophthora infestans: the plant (and R gene) destroyer. Mol Plant Pathol.

[22] Girvan M, Newman MEJ (2002). Community structure in social and biological networks. Proc Natl Acad Sci U S A.

[23] Guelzim N, Bottani S, Bourgine P, Képès F (2002). Topological and causal structure of the yeast transcriptional regulatory network. Nat Genet.

[24] Gyetvai G, Sønderkær M, Göbel U, Basekow R, Ballvora A, Imhoff M (2012). The transcriptome of compatible and incompatible interactions of potato (*Solanum tuberosum*) with *Phytophthora infestans* revealed by DeepSAGE analysis. PloS One.

[25] Haas BJ, Kamoun S, Zody MC, Jiang RHY, Handsaker RE, Cano LM (2009). Genome sequence and analysis of the Irish potato famine pathogen Phytophthora infestans. Nature.

[26] Haury A, Mordelet F, Vera-licona P, Vert J (2012). Open access TIGRESS : trustful inference of gene REgulation using stability selection. BMC Syst Biol.

[27] Hempel S, Koseska A, Nikoloski Z (2013). Data-driven reconstruction of directed networks. Eur Phys J B.

[28] Hu Z, Killion PJ, Iyer VR (2007). Genetic reconstruction of a functional transcriptional regulatory network. Nat Genet.

[29] Huynh-thu A, Irrthum A, Wehenkel L, Geurts P (2010). Inferring regulatory networks from expression data using tree-based methods. PLoS One.

[30] Huynh-Thu VA, Geurts P (2018). dynGENIE3: dynamical GENIE3 for the inference of gene networks from time series expression data. Sci Rep.

[31] Judelson HS (2012). Dynamics and innovations within oomycete genomes: insights into biology, pathology, and evolution. Eukaryot Cell.

[32] Judelson HS, Ah-Fong AM, Aux G, Avrova AO, Bruce C, Cakir C, da Cunha L, Grenville-Briggs L, Latijnhouwers M, Ligterink W, Meijer HJ, Roberts S, Thurber CS, Whisson SC, Birch PR, Govers F, Kamoun S, van West P, Windass J (2008). Gene expression profiling during asexual development of the late blight pathogen Phytophthora infestans reveals a highly dynamic transcriptome. Mol Plant Microbe Interact.

[33] Kamoun S (2006). A catalogue of the effector secretome of plant pathogenic oomycetes. Annu Rev Phytopathol.

[34] Kelley BS, Lee S-J, Damasceno CMB, Chakravarthy S, Kim B-D, Martin GB, Rose JKC (2010). A secreted effector protein (SNE1) from Phytophthora infestans is a broadly acting suppressor of programmed cell death. Plant J.

[35] Kim HD, Shay T, O’Shea EK, Regev A (2009). Transcriptional regulatory circuits: predicting numbers from alphabets. Science.

[36] Kimbrel JA, Di Y, Cumbie JS, Chang JH (2011). RNA-Seq for plant pathogenic bacteria. Genes.

[37] Kinney JB, Atwal GS (2014). Equitability, mutual information, and the maximal information coefficient. Proc Natl Acad Sci U S A.

[38] Lancichinetti A, Fortunato S (2009). Community detection algorithms: A comparative analysis. Phys Rev E.

[39] Lawyer G (2015). Understanding the influence of all nodes in a network. Sci Rep.

[40] Lee S-J, Rose JKC (2010). Mediation of the transition from biotrophy to necrotrophy in hemibiotrophic plant pathogens by secreted effector proteins. Plant Signal Behav.

[41] Lee TI, Rinaldi NJ, Robert F, Odom DT, Bar-Joseph Z, Gerber GK (2002). Transcriptional regulatory networks in Saccharomyces cerevisiae. Science.

[42] Luscombe NM, Babu MM, Yu H, Snyder M, Teichmann SA, Gerstein M (2004). Genomic analysis of regulatory network dynamics reveals large topological changes. Nature.

[43] Marbach D, Costello JC, Küffner R, Vega NM, Prill RJ, Camacho DM (2012). Wisdom of crowds for robust gene network inference. Nat Methods.

[44] Marbach D, Prill RJ, Schaffter T, Mattiussi C, Floreano D, Stolovitzky G (2010). Revealing strengths and weaknesses of methods for gene network inference. Proc Natl Acad Sci U S A.

[45] Marbach D, Schaffter T, Mattiussi C, Floreano D (2009). Generating realistic in silico gene networks for performance assessment of reverse engineering methods. J Comput Biol.

[46] Margolin AA, Nemenman I, Basso K, Wiggins C, Stolovitzky G, Dalla Favera R, Califano A (2006). ARACNE: an algorithm for the reconstruction of gene regulatory networks in a mammalian cellular context. BMC Bioinformatics.

[47] Mendes P, Sha W, Ye K (2003). Artificial gene networks for objective comparison of analysis algorithms. Bioinformatics.

[48] Newman MEJ (2006). Modularity and community structure in networks. Proc Natl Acad Sci U S A.

[49] Newman MEJ (2011). Communities, modules and large-scale structure in networks. Nat Phys.

[50] Parmar K, Blyuss KB, Kyrychko YN, Hogan SJ (2015). Time-delayed models of gene regulatory networks. Comput Math Methods Med.

[51] Peng H, Long F, Ding C (2005). Feature selection based on mutual information: criteria of max-dependency, max-relevance, and min-redundancy. IEEE Trans Pattern Anal Mach Intell.

[52] Pfaffl MW (2001). A new mathematical model for relative quantification in real-time RT-PCR. Nucleic Acids Res.

[53] Roy S, Das D, Choudhury D, Gohain GG, Sharma R, Bhattacharyya DK (2013). Causality inference techniques for in-silico gene regulatory network.

[54] Schaffter T, Marbach D, Floreano D (2011). GeneNetWeaver: in silico benchmark generation and performance profiling of network inference methods. Bioinformatics (Oxford, England).

[55] Schreiber T (2000). Measuring information transfer. Phys Rev Lett.

[56] Singh N, Vidyasagar M (2016). bLARS : an algorithm to infer gene regulatory networks. IEEE/ACM Trans Comput Biol Bioinform.

[57] Sokolova M, Lapalme G (2009). A systematic analysis of performance measures for classification tasks. Inf Process Manag.

[58] Strogatz SH (2001). Exploring complex networks. Nature.

[59] Tompa M, Li N, Bailey TL, Church GM, De Moor B, Eskin E (2005). Assessing computational tools for the discovery of transcription factor binding sites. Nat Biotechnol.

[60] Tung TQ, Ryu T, Lee KH, Lee D. Inferring gene regulatory networks from microarray time series data using transfer entropy. In: Twentieth IEEE International Symposium on Computer-Based Medical Systems (CBMS’07): IEEE; 2007. p. 383–8.

[61] Van Dongen S (2008). Graph clustering via a discrete uncoupling process. SIAM J Matrix An Appl.

[62] Vargas AM, Quesada Ocampo LM, Céspedes MC, Carreño N, González A, Rojas A (2009). Characterization of Phytophthora infestans populations in Colombia: first report of the A2 mating type. Phytopathology.

[63] Wang Z, Gerstein M, Snyder M (2009). RNA-Seq: a revolutionary tool for transcriptomics. Nat Rev Genet.

[64] Yu H, Gerstein M (2006). Genomic analysis of the hierarchical structure of regulatory networks. Proc Natl Acad Sci U S A.

[65] Yuan Y, Li C-T, Windram O (2011). Directed partial correlation: inferring large-scale gene regulatory network through induced topology disruptions. PLoS One.

[66] Zhang M, Lu J, Tao K, Ye W, Li A, Liu X (2012). A Myb transcription factor of Phytophthora sojae, regulated by MAP kinase PsSAK1, is required for zoospore development. PLoS One.

[67] Zoppoli P, Morganella S, Ceccarelli M (2010). TimeDelay-ARACNE: reverse engineering of gene networks from time-course data by an information theoretic approach. BMC Bioinformatics.

