# Supplementary figures and images for: Gene regulatory networks on transfer entropy (GRNTE): a novel approach to reconstruct gene regulatory interactions applied to a case study for the plant pathogen Phytophthora infestans
Source: Theor Biol Med Model. 2019 Apr 9;16:7. doi: 10.1186/s12976-019-0103-7 (PMC6454757; doi:10.1186/s12976-019-0103-7)

BP

400

300

200

100

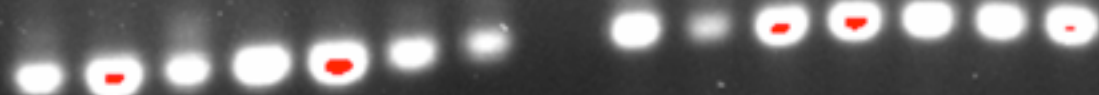

Supplement: Supplementary file 2 — Figure S1. PCR amplification for testing primer viability. cDNA extracted from P. infestans in PDA media was amplified at 45 PCR cycles. Observed fragment size x to expected fragment size (~ 50-70 bp) when observed in 2% agarose gel. Some primer dimers can be observed. (PDF 387 kb) [file 12976_2019_103_MOESM2_ESM.pdf]

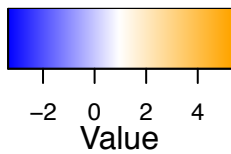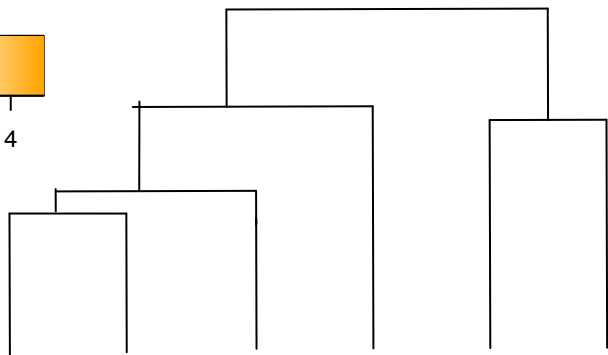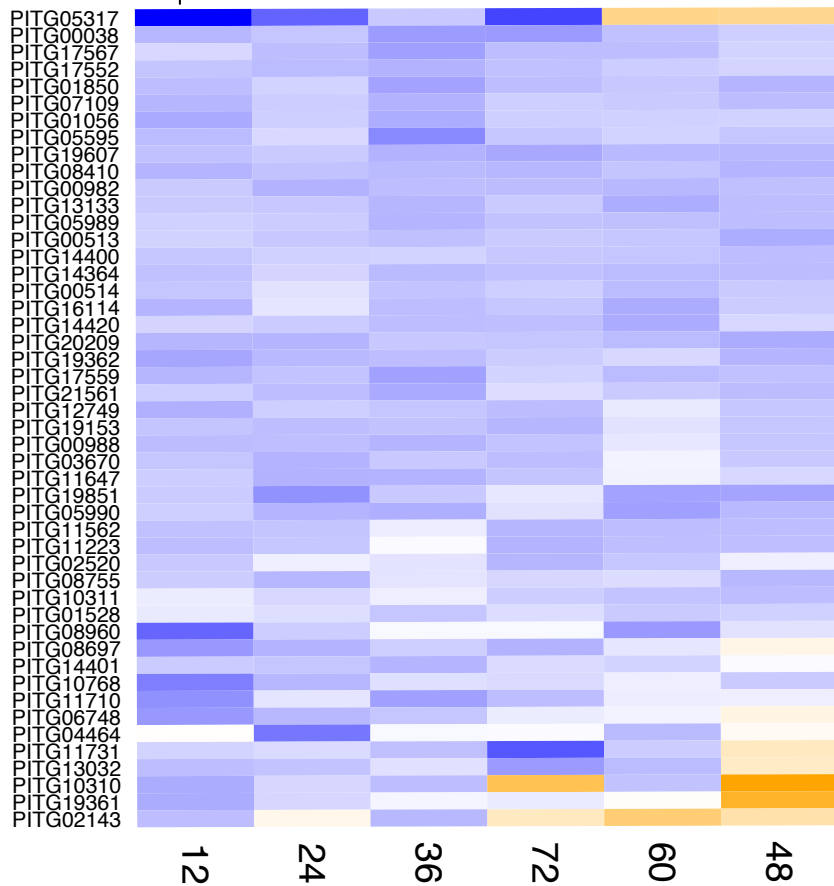

Time (HPI)

Supplement: Supplementary file 4 — Figure S2. Expression profile of Col2 compared to Col3. Heatmap representing the expression profiles were compared for the two cultivars. For each transcript each time point is compared to the same timepoint in the other cultivar (e.g., Col2 PITG_05317 12 h.p.i. is compared to Col3 PITG_05317 12 h.p.i.) Although only minor changes in expression are observed Genes overexpressed in Col2 and a clear separation between early and late infection can be observed by hierarchical clustering. (PDF 189 kb) [file 12976_2019_103_MOESM4_ESM.pdf]

Col2

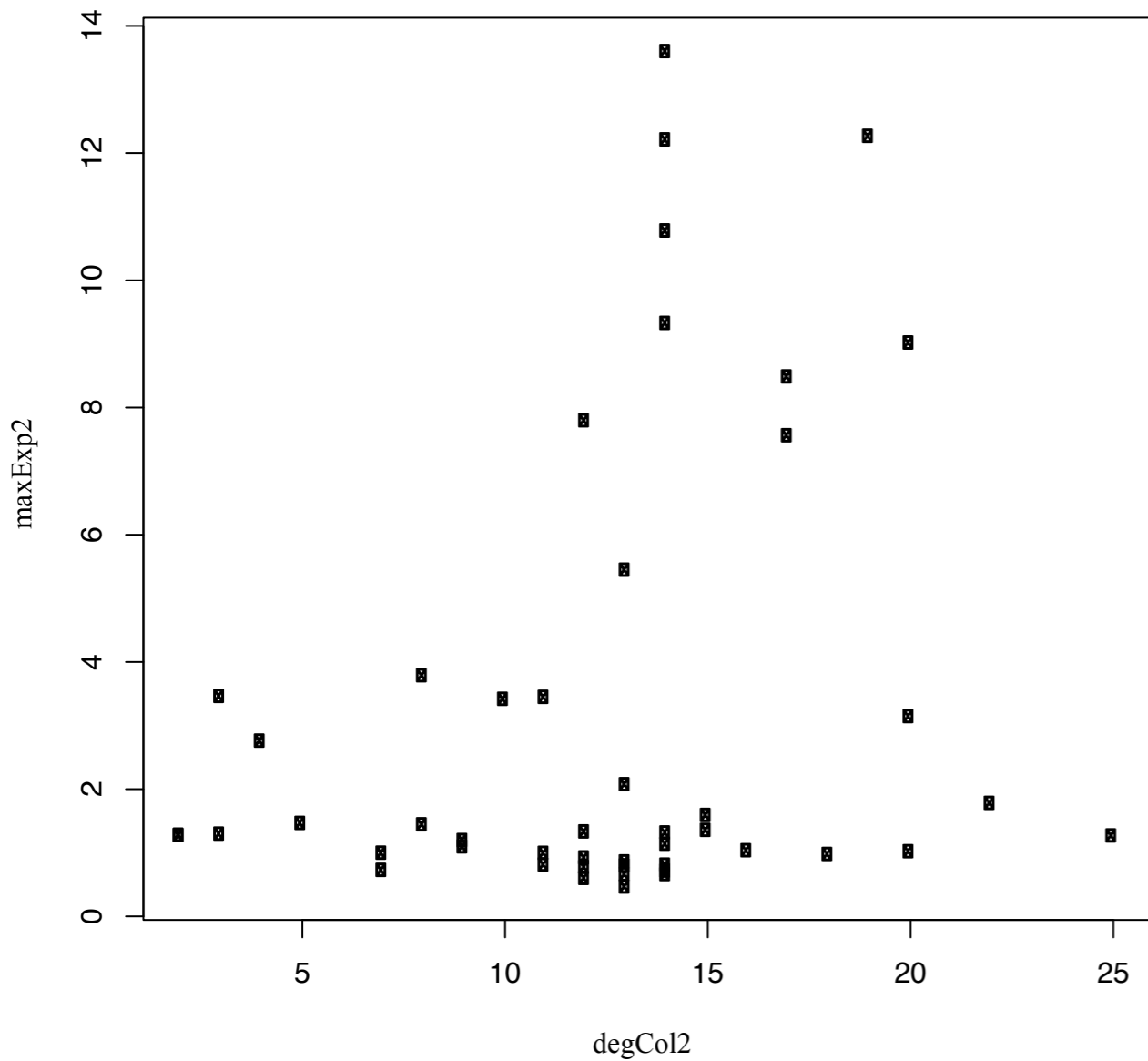

Col3

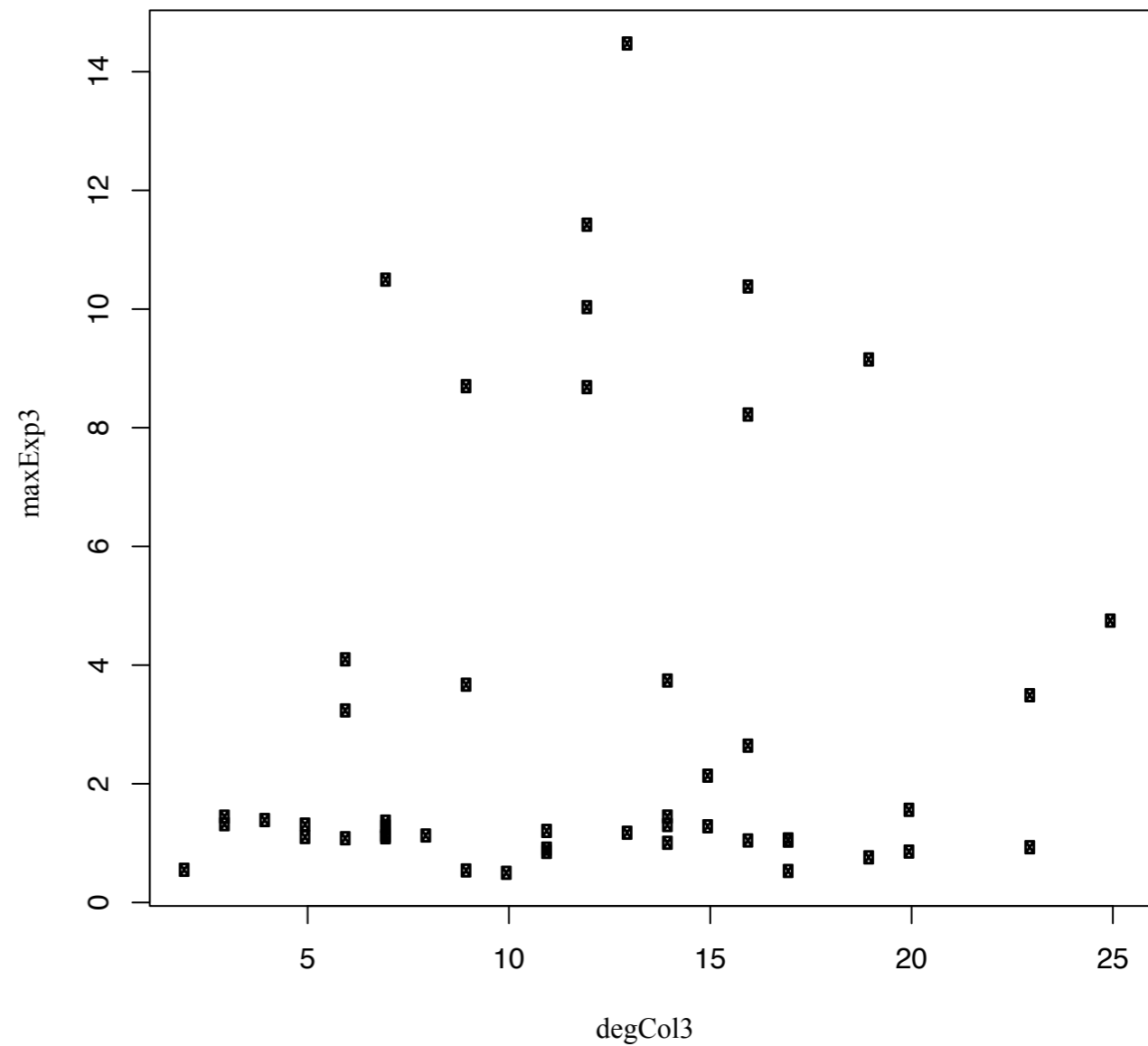

Supplement: Supplementary file 7 — Figure S3. Expression is unrelated to degree. Node degree is computed for each node in the network and plotted against it mean expression value in Col2 (A) and in Col3 (B). No correlation is observed. (PDF 179 kb) [file 12976_2019_103_MOESM7_ESM.pdf]
